# Clinical, Dermoscopic, Ultrasonographic, and Histopathologic Correlations in Kaposi’s Sarcoma Lesions and Their Differential Diagnoses: A Single-Center Prospective Study

**DOI:** 10.3390/jcm12010278

**Published:** 2022-12-29

**Authors:** Athanasia Tourlaki, Gianluca Nazzaro, Yiran Wei, Stefano Buffon, Maria A. Mattioli, Angelo V. Marzano, Lucia Brambilla

**Affiliations:** 1 Dermatology Unit, Fondazione IRCCS Ca’ Granda Ospedale Maggiore Policlinico, 20122 Milan, Italy; 2 Department of Pathophysiology and Transplantation, Università degli Studi di Milano, 20122 Milan, Italy

**Keywords:** Kaposi’s sarcoma, clinical manifestations, dermoscopy, ultrasound imaging, histopathology, differential diagnoses

## Abstract

(1) Background: Kaposi’s sarcoma (KS) is an angioproliferative neoplasm typically appearing as angiomatous patches, plaques, and/or nodules on the skin. Dermoscopy and ultrasonography have been suggested as an aid in the diagnosis of KS, but there is little evidence in the literature, especially regarding its possible differential diagnoses. Our aim is to describe and compare the clinical, dermoscopic, and ultrasonographic features of KS and KS-like lesions. (2) Methods: we conducted a prospective study on 25 consecutive patients who were first referred to our tertiary care center from January to May 2021 for a possible KS. (3) Results: 41 cutaneous lesions were examined by means of dermoscopy, Doppler ultrasonography, and pathology, 32 of which were KS-related, while the remaining 9 were lesions with clinical resemblance to KS. On dermoscopy, a purplish-red pigmentation, scaly surface, and the collarette sign were the most common features among KS lesions (81.3%, 46.9%, and 28.1%, respectively). On US, all 9 KS plaques and 21 KS nodules presented a hypoechoic image. Dermoscopic and Doppler ultrasonographic findings of KS-like lesions, such as cherry angioma, venous lake, glomus tumor, pyogenic granuloma, and angiosarcoma were also analyzed. (4) Conclusions: dermoscopy and Doppler ultrasonography can be useful to better assess the features of KS lesions and in diagnosing equivocal KS-like lesions.

## 1. Introduction

Kaposi’s sarcoma (KS) is an angioproliferative neoplasm first described in 1872 by the Hungarian dermatologist Moritz Kaposi as “idiopathic multiple pigmented sarcoma of the skin”. It presents as violaceous, reddish-blue, or dark brown macules, plaques, and nodules, which may sometimes be frankly exophytic, ulcerative, or hemorrhagic. KS usually affects the distal lower extremities, but more aggressive subtypes can present a diffuse cutaneous distribution as well as mucosal, visceral, and lymph node involvement. KS development is dependent on human herpesvirus 8 (HHV-8) infection, which is therefore noted as Kaposi’s sarcoma-associated herpesvirus [1].

Since its first description, four different epidemiological-clinical variants of KS have been characterized: classic KS occurs in elderly males, generally presenting past the age of 70 years; endemic KS usually affects adult males and children from Equatorial Africa; iatrogenic KS most commonly develops in patients undergoing immunosuppressive therapy after organ transplants; epidemic KS is associated with HIV-induced immunosuppression and generally responds positively to effective antiretroviral treatment regimens [1].

Histopathologic confirmation is generally necessary, as differential diagnosis between KS and other skin disorders, especially vascular neoplasms, can be difficult [2]. While serological assays detecting antibodies to HHV-8 antigens can direct physicians to the diagnosis of KS, HIV serological testing is mandatory to rule out a possible epidemic variant. Additional investigations are useful to define potential mucosal, visceral, or lymph node involvement, as well as to stage KS [2]. As of today, no universally accepted KS staging system is available for clinicians. A staging system articulated on four different stages, based on the clinical heterogeneity of KS lesions and their progressive natural history, has been proposed [3].Therapeutic options depend on the KS stage, with the earliest, indolent stages benefiting from sole clinical observation or topical treatments and those presenting a less indolent disease being candidates for systemic chemotherapy [2,4,5].

Histopathologically, it is useful to consider three distinct stages (patch, plaque, and nodule) which are closely linked to the clinical heterogeneity of KS lesions and their progressive evolution [6]. KS presents as an angioproliferative process located in the dermis in the form of small and irregular vascular spaces surrounded by an inflammatory infiltrate of lymphocytes and plasma cells. Hemosiderin-laden macrophages, free hemosiderin deposits, and eosinophilic hyaline globules can often be located within the newly formed vascular spaces [6,7,8]. From an immunohistochemical standpoint, KS cells express smooth muscle (SMA), dendritic cell (factor XIII), and macrophage (CD68) markers [9]. Therefore, it has been proposed that they may derive from mesenchymal stem cells which underwent a mesenchymal-to-endothelial switch induced by HHV-8 infection [10].

Dermoscopy and ultrasonography (US) can aid in the diagnosis of KS, especially in uncertain cases. On dermoscopy, most KS lesions present a bluish-red pigmentation with a scaly surface and small brown globules in some areas. Interestingly, about one third of KS lesions present the so-called ‘rainbow pattern’ (i.e., close juxtaposition of multiple colors of the rainbow spectrum, from red to violet) [11,12,13]. Bogner et al. first used US to describe KS lesions of six patients suffering from AIDS and compared their relative volumes, thus introducing US as a tool to objectively characterize cutaneous KS [14]. In the following years, more research groups focused their work on the possible application of US and Doppler-mode to advance the general understanding of KS cutaneous lesions, but no standardized description of their different manifestations has yet been validated [15,16]. Considering the limited literature data regarding the full spectrum of KS manifestations, we aim to further illustrate the clinical heterogeneity of KS lesions and describe the correlation existing with their dermoscopic, US, and Doppler-mode appearance based on the experience gathered in our tertiary care center. We additionally present an array of possible clinical, dermoscopic, and ultrasonographic differential diagnoses encountered in these patients.

## 2. Materials and Methods

A prospective case-series study was performed. Men or women aged 18 or over who were primarily referred to our tertiary care center for a possible KS from 1 January, to 31 May 2021 were enrolled. The patients included in our study had recently received a clinical diagnosis of possible KS in other centers and had one or more skin lesions resembling KS, thus needing diagnostic confirmation. Recruitment was on a consecutive basis and the study was performed in accordance with the departmental guidelines. As the established routine management of patients was not altered, a specific ethical committee approval was deemed unnecessary. All the participants signed an informed consent form, agreeing to the execution of skin biopsy, serology testing for HIV and HHV-8, as well as publication of clinical photographs. The only exclusion criterion was a history of biopsy-proven KS, since a new biopsy could be unnecessary. 

Patients’ demographic characteristics, medical history, prior and current treatments were recorded. Cutaneous KS staging was according to the staging system of Brambilla et al. [2].

Clinical photographs were taken with a Nikon D5100 digital camera. Dermoscopic examination was accomplished thanks to either a bodystudio ATMB® Master (FotoFinder, Teachscreen Software GmbH, Bad Birnbach, Germany) videodermatoscope or a manual Dermlite DL4 dermatoscope (3Gen Inc., San Juan Capistrano, CA, USA), with the employment of ultrasound gel when necessary to avoid pressure-induced compression of vascular structures. Evaluation of the dermoscopic images was performed by two of the authors (G.N., A.T.) in consensus; if no consensus was obtained, the according feature was scored as absent.

The US platform implemented by one of us (G.N.) was the Hitachi Arietta V850 with multifrequency linear array transductor (15.0–18.0 MHz). Ultrasonography parameters analyzed the depth and margins of the lesions, echogenicity, and vascularity defined according to Giovagnorio’s criteria [17]. More specifically, the tumors were classified as: avascular (type I); hypovascular with a single vascular pole (type II); hypervascular with multiple peripheral poles (type III); hypervascular with internal vessels (type IV).

Biopsies were performed in all lesions examined. Hematoxylin-eosin stains were employed and immunohistochemical markers (HHV-8, CD32, CD34, D2-40) were tested.

## 3. Results

Twenty-five consecutive patients were enrolled in the present study; 18/25 (72%) patients were male, and 7/25 (28%) female, with a mean age of 73±10.5 years. Twenty-three (92%) patients received a diagnosis of KS, while in the remaining 2 (8%), although there was a clinical resemblance to KS, the final diagnosis was nodular melanoma and Merkel cell carcinoma, respectively. Most of the patients suffered from classic KS (19/23, 82.6%), while cases of epidemic and endemic KS amounted to 2 each. As far as the stage at the moment of diagnosis was concerned, 4 patients (17.4%) were at stage I, 2 (8.7%) at stage II, 3 (13%) at stage III, and 14 (60.9%) at stage IV (Table 1).

We analyzed a total of 41 lesions, 32 (78%) of which were identified as KS. The remaining 9 (22%) were lesions referred to our center as potential KS, but receiving a different histopathological diagnosis. Of the 32 KS lesions, 2 (6.2%) were patches, 9 (28.1%) were plaques, and 21 (65.7%) were nodules. The 9 non-KS lesions included 1 cherry angioma, 3 venous lakes, 1 glomus tumor, 1 pyogenic granuloma, 1 angiosarcoma, 1 nodular melanoma, and 1 Merkel cell carcinoma.

Analyzing the 32 KS lesions usingdermoscopy, a purplish-red pigmentation was observed in 26 (81.3%) of them, while a scaly surface and the collarette sign were appreciable in 15(46.9%) and 9 (28.1%) lesions, respectively. The rainbow pattern was present in 8 (25%) lesions, 7 of which were nodules. As far as vascular architecture was concerned, the most frequent pattern was serpentine (9/32, 28.1%), followed by dotted and curved/coiled (5/32 (15.6%) and 3/32 (9.4%), respectively). Table 2 reports the frequency of these dermoscopic features in non-KS lesions. Being for the most part of a vascular nature, they mainly exhibited a bluish-reddish pigmentation (6/9, 66.6%).

On US examination, all 9 KS plaques and 21 KS nodules presented a hypoechoic image. The Doppler-mode signal was positive in 1 plaque and 20 nodules, with a lower pole distribution in 16/21 (76.1%). In parallel, all 9 non-KS cutaneous lesions presented a hypoechoic image, while Doppler-mode examination was positive in only 5 of them (Table 3).

### 3.1. Patches

The active patch presented as a flat, reddish-violaceous lesion with well-defined margins, and some areas showing a scaly surface. Dermoscopy showed a structureless, homogeneously pigmented violaceous area, crossed by fine branching vessels, which were more evident at the periphery of the lesion. On the other hand, the remitting patch was characterized by a pinkish-brown coloration, sharp though irregular margins, and absence of inflammatory features. Dermoscopic examination showed small blotches of rose-colored or light brown pigmentation with a reticular distribution, interspersed with normal-colored healthy skin. Vessels were not prominent.

**Figure 1:**
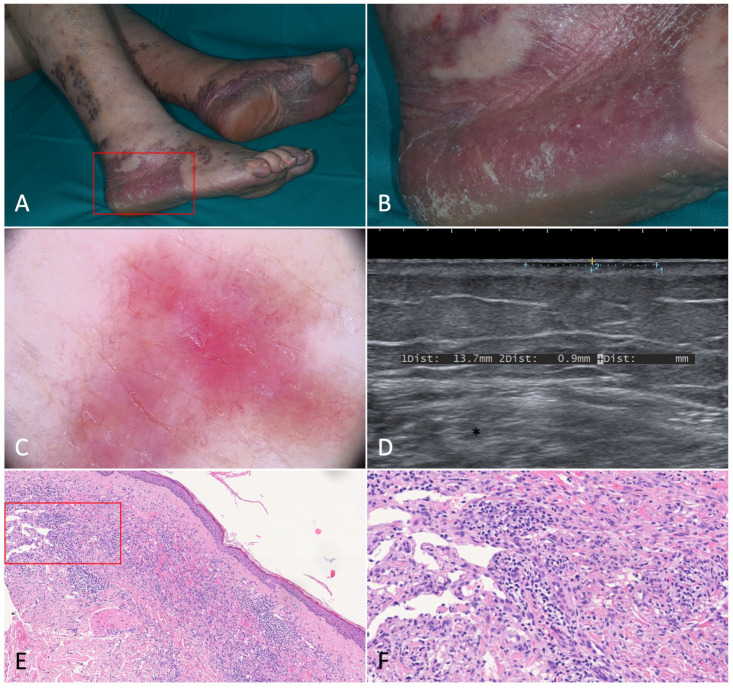
Patch stage Kaposi’s sarcoma. (**A**,**B**). Active patches on the feet of a 79-year-old female patient suffering from classic Kaposi’s sarcoma. (**C**). Dermoscopy of one of the patches revealed a homogeneously structureless pinkish-brown area crossed by fine branching vessels. (**D**). Ultrasonography showed a thin hypoechoic linear band in the superficial dermis. (**E**). Histologically, the lesion was characterized by a dermal proliferation of spindle cells forming sinuous vascular spaces and a lymphocytic infiltrate [Hematoxylin-eosin, 40×]. (**F**). Higher magnification revealing the promontory sign (*) with protrusion of vascular structures into lumens of the few newly formed vascular spaces [H-E, 100×].

US and Doppler examination of the two KS patches in our patients revealed a signal which was identical to that of the surrounding tissues. No difference was found between the clinically active and the remitting lesion since they were both completely comparable to normal skin as far as their US features were concerned (Figure 1).

### 3.2. Plaques

Active KS plaques presented a violaceous-brown pigmentation, revealing the underlying vascular proliferation, and increased consistency as a result of significant tumor infiltration. Like patches, their margins appeared sharp. On skin areas characterized by a prominent stratum corneum, such as the feet, plaques showed a frankly hyperkeratotic aspect and were surmounted by variably thick, tightly adherent white scales. Dermoscopically, whitish scaly areas and scattered brown blotches were visible on a bluish-purple background with irregular but well-demarcated borders. Dotted vessels appeared more prominent at the edge of the lesions.

When compared to active plaques, remitting ones were less obviously angiomatous in appearance and had a brownish pigmentation. Infiltration tended to subside alongside lesion thickness. From a dermoscopic standpoint, a central area of homogenous dark blue pigmentation was surrounded by a brown regular network, which tended to fade and become less organized at the periphery of the lesion. The network was interspersed with areas of normal skin pigmentation, which gave the lesion an irregular appearance.

**Figure 2:**
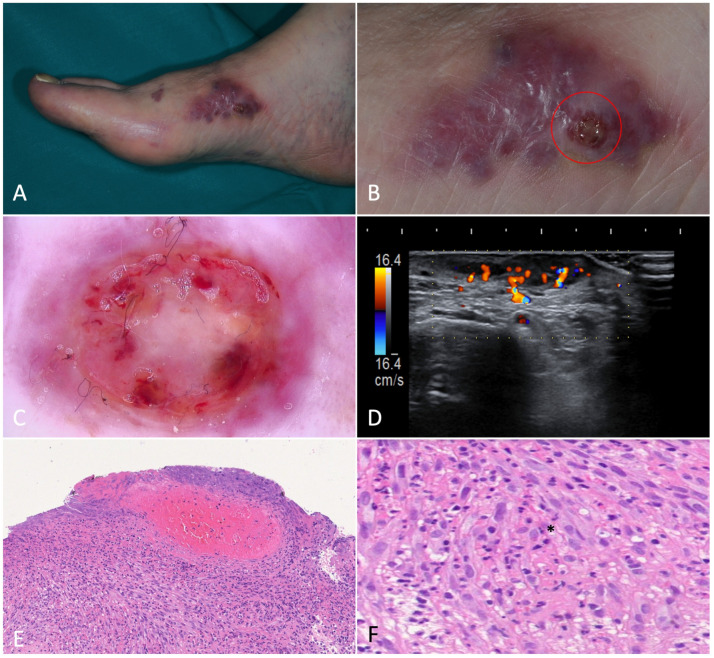
Bullous Kaposi’s sarcoma. (**A**). Angiomatous plaque on the right foot medial surface of a 75-year-old male patient with classic Kaposi’s sarcoma. (**B**). Magnification of the area showing confluent bullae forming a plaque. (**C**). Dermoscopy of a bulla showing a target-shaped lesion with whitish, purple and red concentric, structureless areas and short dilated vessels in the periphery. (**D**). Ultrasonography revealed a homogenous, hypoechoic lesion in the dermis and subcutis. Color-Doppler showed intense intralesional and deep vascular flow. (**E**). Histological examination showed dilated interstitial spaces delimited by bundles of spindled cells in association with erythrocyte extravasation in the dermis (H-E, 20×). (**F**). Hemorrhagic-necrotic material in the upper part of the image (H-E, 40×). H. At higher magnification we can appreciate the phenomenon of autolumination (*): the presence of an erythrocyte within a paranuclear vesicle in one spindle cell (H-E, 200×).

A peculiar variant observed was the so-called bullous KS, which consists of the development of lymph-filled compressible vesiculobullous lesions, usually in the context of a KS plaque. Dermoscopically, the plaque in our study presented a violaceous-blue homogeneous pigmentation with short, tortuous vessels. The single bulla showed a clear central portion, surrounded by angiomatous or frankly hemorrhagic blotches and dilated irregular vessels (Figure 2).

On US, all KS plaques examined were identifiable as a hypoechoic band without any vascular signal in Doppler-mode, except for the pseudobullous plaque. Hyperkeratosis was detected as a thickening of the stratum corneum. When comparing US features of clinically active and inactive plaques, no difference was observed.

### 3.3. Nodules

**Figure 3:**
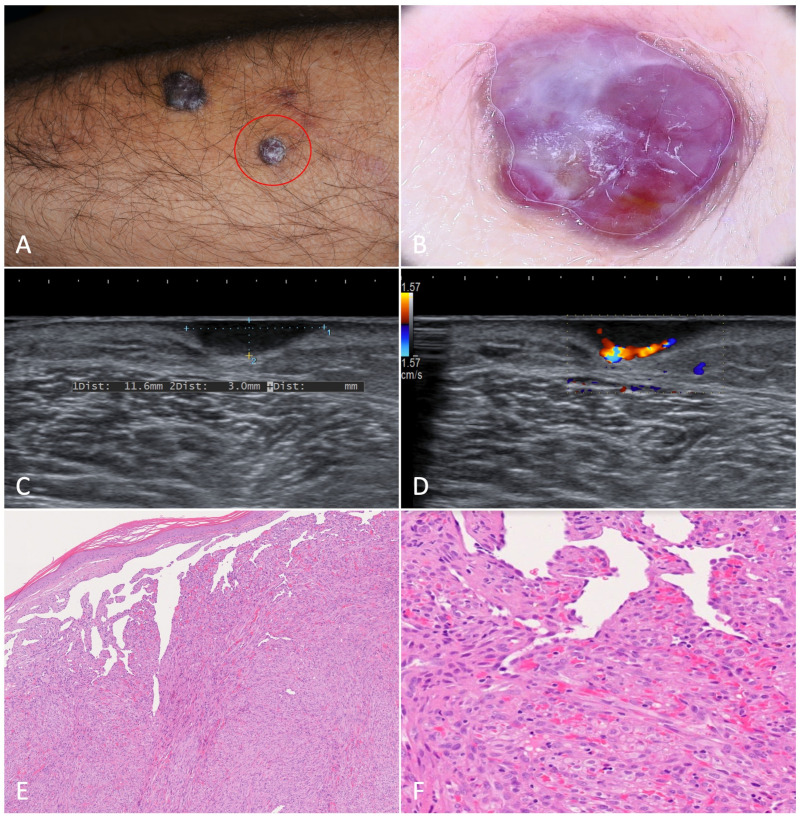
Nodular Kaposi’s sarcoma. (**A**). Hyperkeratotic nodules on the left forearm of an 81-year-old patient suffering from classic Kaposi’s sarcoma. (**B**). Dermoscopy showed a purplish nodule with whitish-yellow collarette. The rainbow pattern can be clearly seen. (**C**,**D**). A hypoechoic nodular lesion, characterized by vascular signal detected with Color-Doppler at the lower pole. (**E**). Histology showed fascicles of spindle cells forming sinuous vascular spaces in the dermis, especially towards the periphery of the lesion (H-E, 20×). (**F**). Higher magnification revealed eosinophilic hyaline globules as a result of the autolumination process (H-E, 100×).

**Figure 4:**
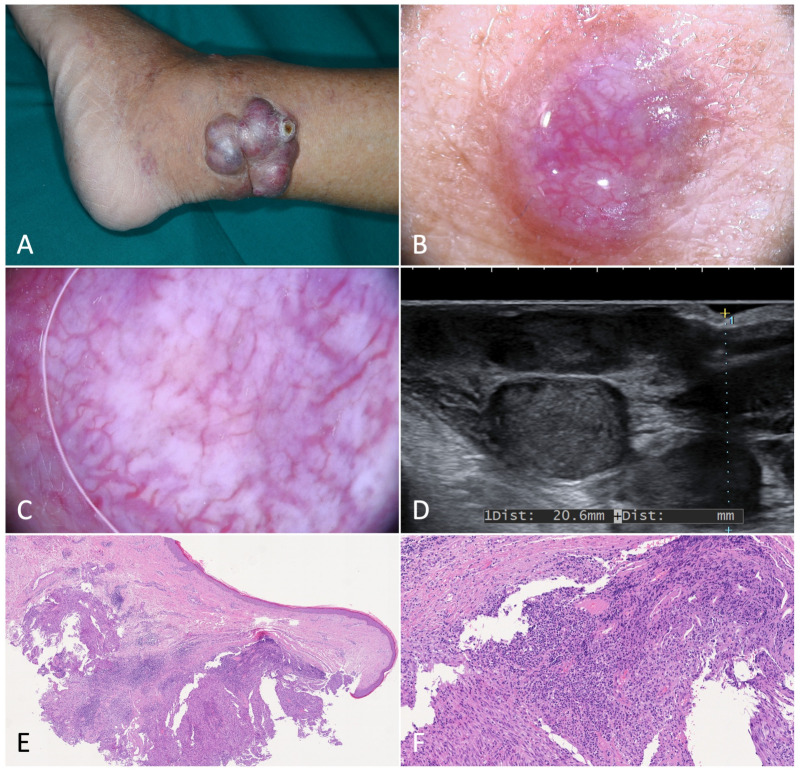
Exophytic nodular Kaposi’s sarcoma. (**A**). Exophytic nodules on the left ankle of an 84-year-old male patient diagnosed with classic Kaposi’s sarcoma. (**B**). Magnification of a translucent purplish-red nodule characterized by telangiectasias. (**C**). Dermoscopy showed dilated, serpentine vessels, homogeneously distributed across the nodule. (**D**). B-mode ultrasonography showed multiple nodules divided by septa and characterized by inhomogeneous hypoechogenicity. (**E**,**F**). Histology revealed a well-circumscribed dermal mass formed by monomorphic spindled cells organized in bundles and delimited by dilated confluent vascular spaces (H-E, 20× and 80×).

Single nodules ranged from a few millimeters to more than a centimeter in diameter. Active nodules were characterized by an intense purple pigmentation, which is a sign of underlying active vascular proliferation, and often appeared surmounted by white scales. They were delimited by a hyperkeratotic collarette dividing them from the normal-appearing surrounding skin. Dermoscopic examination showed a sharply demarcated violaceous lesion crossed by mostly linear dilated vessels. When present, the rainbow pattern was seen extensively throughout the whole nodule (Figure 3). Sometimes nodules were confluent and/or frankly exophytic (Figure 4).

Hyperkeratotic nodules arose mostly on palms and soles, where the stratum corneum is particularly thick. On dermoscopic examination, a peripheral hyperkeratotic collarette appeared to surround a central area of brownish pigmentation. White dots were detectable at the opening of the pilosebaceous unit. No vascular structures were visible.

Some nodules developed in the context of an angiomatous plaque. On dermoscopy they showed similar features to those previously described for single nodules, mainly white scaling with a peripheral collarette and a violaceous central area crossed by irregular vessels. Examination of the plaque revealed a flat lesion with a fading reddish-purple pigmentation at the periphery, and central milky structureless areas. Hemosiderin deposition was appreciable as areas of yellowish pigment.

**Figure 5:**
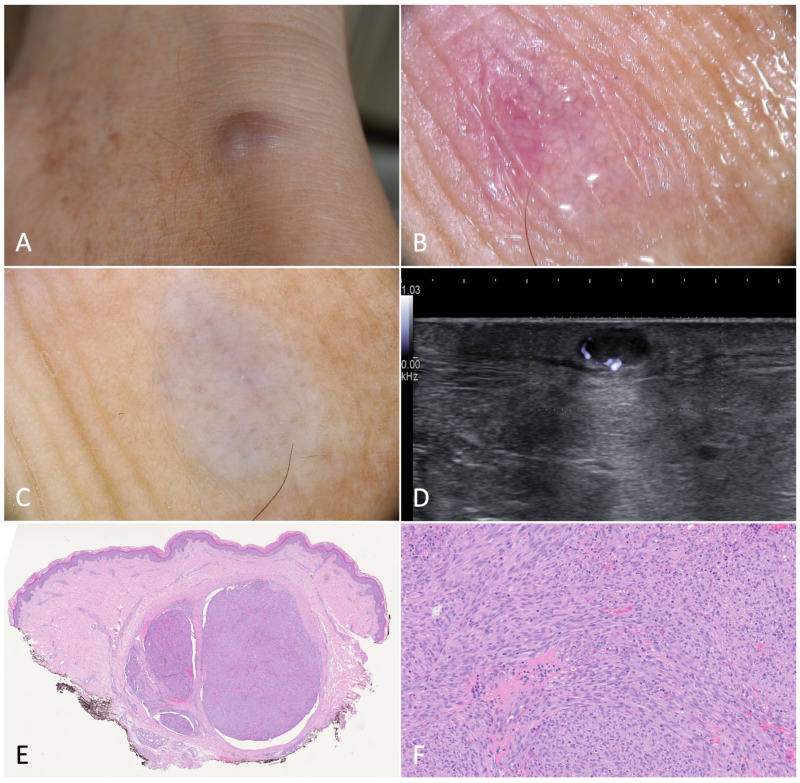
Deep nodular Kaposi’s sarcoma. (**A**). Deep nodule on the right heel of a 61-year-old male patient suffering from classic Kaposi’s sarcoma. (**B**). Dermoscopy without applying pressure showed serpentine vessels with a reticulated distribution. (**C**) Dermoscopy with downward pressure caused the emptying of the vessels. (**D**). B-mode ultrasonography revealed a hypoechoic nodule with posterior enhancement. (**E**). Histology showed some grouped nodules deeply localized in the dermis, and separated by fibrous septa (H-E, 20×). (**F**). Spindled cells arranged in haphazard fascicles. Intra- and extracellular hyaline globules were detectable (H-E, 100×).

Rarely, KS nodules were localized deep in the subcutaneous tissue, with or without overlying visible skin changes (Figure 5). These nodules were soft to firm in consistency and non-tender to palpation, not freely movable in relation to the underlying tissue. In our study, dermoscopy of subcutaneous KS nodules was highly non-specific, showing a lesion with undefined margins and a similar pigmentation to that of the surrounding skin or a bluish-pink structureless area.

As far as US examination was concerned, 19 out of 21 KS nodules demonstrated vascular signals at varying speeds. For descriptive purposes, the KS nodular lesions examined have been divided into 5 groups: single nodules, hyperkeratotic nodules, multiple nodules, nodules on plaque, and deep nodules (Table 3).

## 4. Dermoscopic and Ultrasonographic Differential Diagnoses

### 4.1. Cherry Angioma

Cherry angiomas are asymptomatic bright red, blue, or purple dome-shaped papules, mostly located on the trunk or proximal extremities. On dermoscopy, they exhibited small, well-demarcated red-purple-bluish areas (‘lagoons’), corresponding to dilated dermal capillaries. Such lagoons were often separated by whitish septa. US imaging of cherry angiomas revealed thin, hypoechoic, and superficial lesions, since they were limited to the upper dermis. No vascular flow was detectable through Doppler analysis.

### 4.2. Venous Lake

Venous lakes tend to develop on the face, lips, and ears of older patients. As they depend on dilation of venous capillaries, the characteristic bluish pigmentation fades when the lesion is compressed. Dermoscopy revealed purple-bluish structureless and inhomogeneous areas with no identifiable vascular structures. US imaging showed only an intradermal hypoechoic area, and no signs of blood flow were detectable through Doppler-mode. In some venous lesions, theorized to be dependent on size, it was possible to distinguish a multilobular structure in which anechoic spaces were separated by hypoechoic septa, recalling their histopathological features.

### 4.3. Glomus Tumor

**Figure 6:**
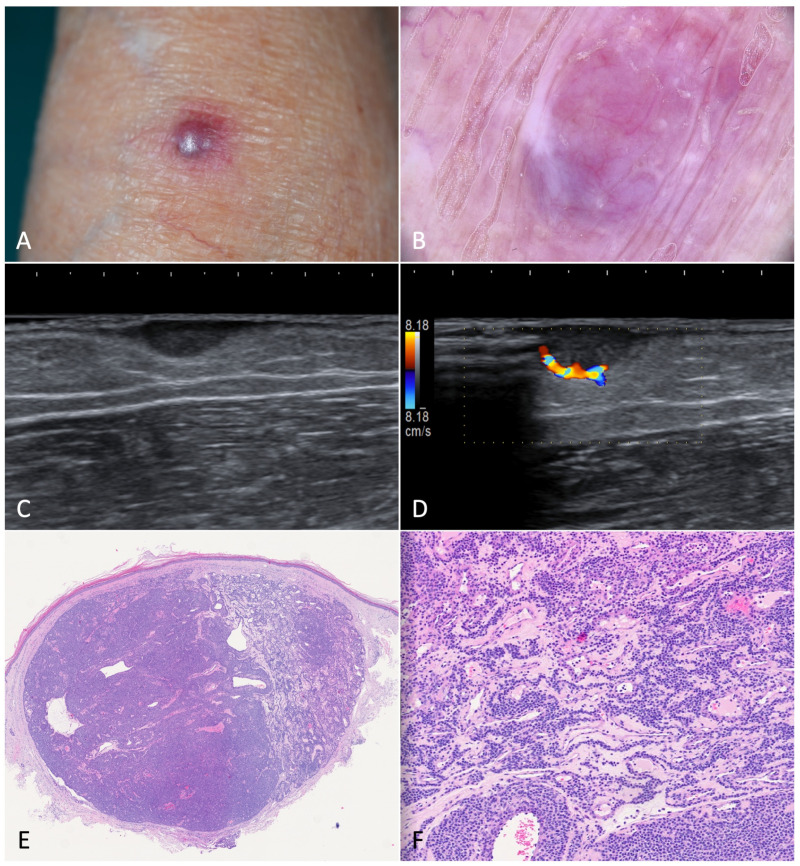
Glomus tumor. (**A**). A painful nodule located on the left forearm of an 85-year-old woman suffering from classic Kaposi’s sarcoma. (**B**). Dermoscopy showed a purple-pinkish nodular lesion characterized by arborescent vessels. (**C**,**D**). B-mode ultrasonography revealed a horizontally oriented, hypoechoic mass with clear borders. On the left, we can see the afferent artery with the so-called stalk-sign. (**E**). Histopathology revealed a mixed eosinophilic and basophilic well-defined mass (H-E, 20×). (**F**). At higher magnification, the specimen was characterized by round glomus cells with pale eosinophilic cytoplasm. Tumor stroma appeared myxoid and edematous (H-E, 80×).

These rare cutaneous tumors usually arise in areas rich in glomus bodies, such as the palm, wrist, forearm, and foot, as well as the subungual region of fingers. Clinically, they present as pink or purple vascular papules or nodules, and the patient often complains of tenderness and cold- or touch-induced paroxysmal pain. In our case, a pink, ‘shiny’ nodule with an erythematous peripheric halo and branching vessels was visible on dermoscopy. US imaging revealed a hypoechoic nodular area with a blood flow detectable through Doppler-mode, mainly distributed at the periphery of the lesion. On histopathology, the lesion was composed of inhomogeneously distributed vascular and cellular proliferating components (Figure 6).

### 4.4. Pyogenic Granuloma

**Figure 7:**
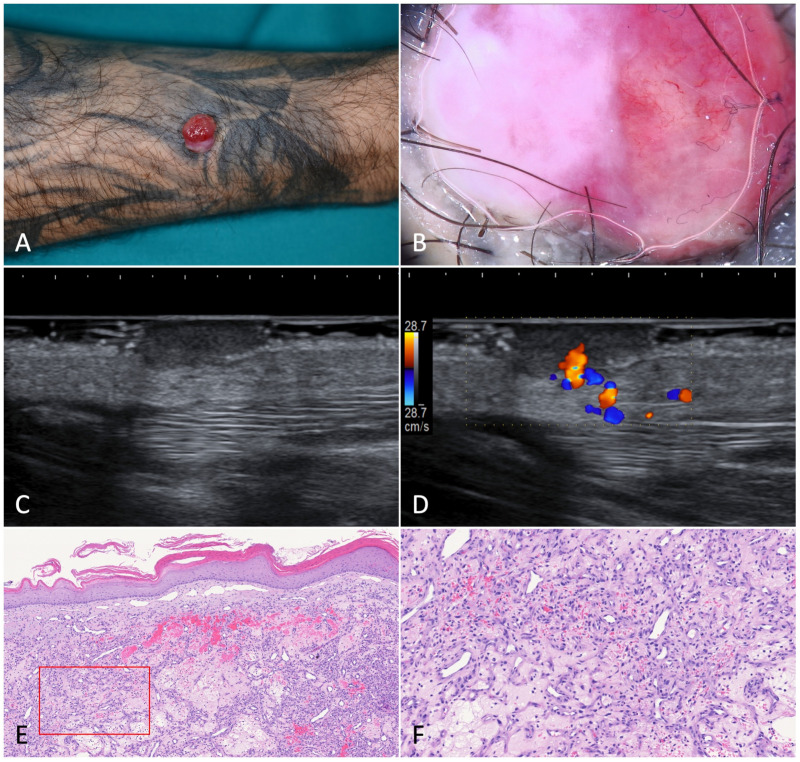
Pyogenic granuloma. (**A**). A pedunculated, angiomatous, rapidly growing nodular lesion on the right forearm of a 44-year-old male patient suffering from HIV-associated Kaposi’s sarcoma. (**B**). Dermoscopy of the nodule was characterized by homogeneous white-reddish areas with numerous linear-irregular vessels. Blanching of the blood vessels due to the pressure applied on the skin surface is shown in the left part of the nodule. (**C**). Ultrasonography showed an inhomogeneous, hypoechoic, and capsulated oval mass. (**D**). Color-Doppler analysis revealed a highly vascularized peduncle (speed 28.7 cm/s). (**E**). Histology showed discontinuous parakeratinized stratified squamous epithelium and vascular spaces in the underlying stroma; (**F**). The nodule did not present fibrous septa, but a fibrinopurulent membrane on the periphery (H-E, 20× and 80×).

Pyogenic granuloma is a solitary benign vascular tumor characterized by rapid growth and a friable surface, thus bleeding easily. In our study, dermoscopy highlighted the structureless homogeneous areas with a white-reddish pigmentation crossed by linear-irregular capillaries. When investigated through US and Doppler-mode, a solid, hypoechoic nodular lesion with high-velocity vascular pedicle was identified. Histological examination revealed proliferating small blood vessels delimited by endothelial cells and edematous stroma (Figure 7). 

### 4.5. Angiosarcoma

Angiosarcoma is a soft tissue sarcoma appearing as bluish or red ulcerated nodules, usually on the head and neck. In our case, the angiosarcoma was located on the arm. On dermoscopy, it presented as a violaceous nodule with a central ulcerated portion, partially covered by brownish hematic crusts. At the periphery of the lesion, vascular lagoons of various size and shape, as well as polymorphous capillaries, were seen. On sonographic examination, angiosarcomas and KS nodular lesions are virtually indistinguishable, since both are usually localized in the dermis and associated with an inhomogeneous and hypoechoic returning signal. On the other hand, Doppler examination revealed mostly peripheral vascularization. Histologically, our specimens showed proliferating epithelioid, pleomorphic, and spindle cells, in addition to multifocal vascular elements.

### 4.6. Nodular Melanoma

Nodular melanoma corresponds to a melanoma in the vertical growth phase and usually appears as a darkly pigmented, sometimes ulcerated, pedunculated or polypoid nodule. In our case, dermoscopic examination revealed an ulcerated area, bleeding at the center, surrounded by a darker, black-brownish peripheral rim with pseudopods. No clear vascular structures or patterns were visible on dermoscopy. Conversely, on US and Doppler-mode examination, the nodular melanoma appeared as an ill-defined, hypoechoic, and prominently vascularized lesion with more than one vascular peduncle.

### 4.7. Merkel Cell Carcinoma

Merkel cell carcinoma is a rare and aggressive neuroendocrine cutaneous neoplasm usually affecting older individuals. It generally presents as a rapidly growing pink-reddish or violaceous nodule with a firm consistency. In our case, dermoscopy showed a chaotic architecture, with milky pink areas and structureless whitish areas, crossed by linear and/or polymorphous capillaries and pinpoint red dots. No pigmentation was visible. US and Doppler-mode examination highlighted a dermal-hypodermal anechoic mass with hypoechoic digitiform projections at the lower pole. Moreover, a prominent intralesional vascularization with a mainly vertical arrangement of the vessels was observed. Histopathological analysis showed a trabecular lesion associated with necrotic areas and dilated capillaries.

## 5. Discussion

Consistent with available data in the literature, dermoscopy in our patients revealed how a bluish-red-purple pigmentation was the most common dermoscopic feature in KS [11,12]. Such clinical presentation was detectable in all patches, and most KS plaques and nodules. Unsurprisingly, a similar coloration was appreciable in several non-KS nodular lesions, in particular cherry angioma, venous lake, pyogenic granuloma, and angiosarcoma, indicating that such a characteristic is not specific for KS, being attributable only to the vascular nature of the lesion.

The rainbow pattern was found infrequently, consistent with previous publications [13,18]. Among the non-KS nodules, no lesion presented this pattern; however, we are disinclined to consider it a pathognomonic dermoscopic finding of KS based upon the limited quantity of lesions examined for each possible differential diagnosis. In fact, data from larger cohorts in the literature have reported the rainbow pattern in other conditions including angiokeratoma, stasis dermatitis, pseudo-Kaposi, melanoma, basal cell carcinoma, and cutaneous scars [19,20,21,22]. In particular, Vázquez-López et al. hypothesized that the ‘rainbow phenomenon’ may emerge due to the interaction of light in different states of polarization, with superficial and deep structural elements of the observed lesion [20]. Therefore, instead of representing a pathognomonic feature of KS, it is dependent on the proportion of vascularization within the lesion. Histopathological examination of lesions presenting the rainbow pattern revealed minimal stromal tissue, with few spindle and endothelial cells surrounding numerous closely packed capillaries with wide vascular lumina forming a honeycomb-structured pattern [11,12]. Similar histopathologic findings characterized by a peculiar vascular architecture within non-KS lesions might also be associated with dermoscopic evidence of the rainbow pattern.

A scaly surface and the collarette sign were detected in a considerable number of KS lesions, mostly in nodules. Histologically, they correspond to focal hyperkeratosis with variable thickening of the stratum corneum and presence of a pseudocapsule in KS nodules. Among the non-KS nodular lesions examined, none presented such features.

Dermoscopy of cutaneous lesions with clinical resemblance to KS was consistent with the literature. More specifically, cherry angiomas are mostly described as rounded lesions showing red lacunae separated by fibrous septa [23]. Venous lakes present reddish-blue to purple structureless areas or globules, occasionally associated with white structures when examined through dermoscopy or mucoscopy [24,25]. The main dermoscopic features of pyogenic granuloma comprise reddish homogeneous areas with a white collarette and the so-called “white rail lines”. Linear-irregular vessels are reported as frequent dermoscopic features as well [26]. Dermoscopy of cutaneous angiosarcomas reveals pink-purple areas known as “steam-like areas”, characterized by a white or skin-colored central area and peripheral purple enhancement [27]. Extradigital glomus tumors are mostly described as lesions with pink structureless areas, without lacunae, as opposed to other vascular tumors [24,28]. Lastly, the most common dermoscopic features of Merkel cell carcinoma include milky-red areas and polymorphous vascular pattern, mainly consisting of linear-irregular and dotted vessels [29].

In our findings, US analysis was unable to detect patches, whilst KS plaques were identifiable with a hypoechoic band without any vascular flow on Doppler-mode.

When examined on US, KS nodules appeared hypoechoic, mostly localized within the dermis, with well-defined margins corresponding to a pseudocapsule. The minor US waves reflection of KS nodules, when compared to the surrounding tissues, explains the observation of a posterior acoustic enhancement. Interestingly, when analyzing hyperkeratotic nodules, the thickening of the stratum corneum was detectable on US. In most of the KS nodules included in our study, US and color Doppler analysis evidenced a typical vascular distribution at the inferior pole of the lesion. This finding was consistent with the literature and histological results [15]. 

US and Doppler-mode examination of KS cutaneous lesions was recently investigated by Solivetti et al. The authors have compared US imaging results obtained from classic and epidemic KS lesions, concluding that a vascular signal was more frequently detectable in the latter group than in the former [16]. In our study, nodules showed higher blood flow velocities when compared to patches and plaques. In fact, among the lesions we considered, all KS nodules, except for a hyperkeratotic one, showed detectable blood flow on Doppler examination, whilst only one plaque showed Doppler signs of vascular flow. This finding may be related to the prominent endothelial proliferation within KS nodules on histology compared to other KS lesions [7]. Doppler analysis has already proven valuable in evaluating the vascularity of KS lesions, since a progressive reduction of the caliber of the deepest pole vessels after vincristine injections was observed to predict nodule remission [30,31]. Hence, even though further studies are required to draw final conclusions, US and Doppler analysis could be a useful tool to characterize KS cutaneous lesions activity and disease progression. The aforementioned techniques could likewise be useful in identifying a deep localized KS lesion which cannot be clinically diagnosed with certainty [32].

Ultimately, US and Doppler analysis may be fundamental in the process of differential diagnosis of cutaneous nodular lesions. This is especially true for those patients who have received a KS diagnosis, thus avoiding unnecessary excision of suspect lesions, alongside considering the tendency of KS to recur upon surgical wounds [33,34].

Although data regarding sonographic and Doppler appearance of cutaneous vascular lesions are often not univocal, some distinctive markers are consistent with our results. All nodular lesions examined appeared as hypoechoic areas, which represents a nonspecific finding for cutaneous vascular lesions. Contrary to the cherry angioma and venous lakes, the remaining lesions showed identifiable blood flow on Doppler examination. Some lesions were characterized by a peculiar pattern different from that of KS nodules, in which vascularization was predominantly localized at their lower pole.

Regarding the sonographic appearance of the non-KS lesions, our findings were consistent with other published literature [35,36,37,38,39,40,41].

The main limitation of our study was the small sample size, especially regarding the number of non-KS lesions, which did not allow us to perform a statistical analysis. The small sample size may also explain some possible discrepancies between our dermoscopic and US findings compared to the data reported in the literature.

## 6. Conclusions

Dermoscopy, US, and Doppler analysis can be useful tools in helping clinicians in the differentiation between KS and non-KS skin lesions. This is especially valuable when uncommon nodular lesions arise in patients with a previous diagnosis of KS. While the use of dermoscopy is expanding, further studies are required to determine the extent and applicability of the US imaging and Doppler analysis within daily clinical practice.

## Figures and Tables

**Table 1:** Data regarding the 25 patients enrolled in our study.

Gender M F	18 (72%) 7 (28%)
Mean age	73 ± 10.5 y
KS confirmation Yes No	23 (92%) 2 (8%)
KS variant Classic Epidemic Endemic	19 (82.6%) 2 (8.7%) 2 (8.7%)
KS stage at diagnosis I II III IV	4 (17.4%) 2 (8.7%) 3 (13%) 14 (60.9%)
Cutaneous lesions analyzed KS lesions Non-KS lesions	41 32 (78%) 9 (22%)

**Table 2:** Frequency of dermoscopic features among KS and non-KS lesions (see horizontal page at the end of the article).

	Purplish-Red Pigmentation, n (%)	Rainbow Pattern, n (%)	Scaly Surface, * n (%)	Collarette Sign *, n (%)	Vascular Architecture, n (%)		
					Serpentine	Dotted	Curved/Coiled
KS lesions							
Patch (2)	2 (6.3)	-	-	-	1 (3.1)	1 (3.1)	0
Plaque (9)	7 (21.9)	1 (3.1)	6 (18.8)	-	3 (9.4)	1 (3.1)	1 (3.1)
Nodule (21)	17 (53.1)	7 (21.9)	9 (28.1)	9 (28.1)	5 (15.6)	3 (9.4)	2 (6.3)
Total (32)	26 (81.3)	8 (25)	15 (46.9)	9 (28.1)	9 (28.1)	5 (15.6)	3 (9.4)
Non-KS lesions							
Cherry angioma (1)	1 (11.1)	-	-	-	-	-	-
Venous lakes (3)	3 (33.3)	-	-	-	-	-	-
Glomus tumor (1)	-	-	-	-	1 (11.1)	-	-
Pyogenic granuloma (1)	1 (11.1)	-	-	-	1 (11.1)	-	-
Angiosarcoma (1)	1 (11.1)	-	-	-	1 (11.1)	-	1 (11.1)
Nodular melanoma (1)	-	-	-	-	-	-	-
Merkel cell carcinoma (1)	-	-	-	-	1 (11.1)	1 (11.1)	-

* 17/21 (81%) of the nodules were present on the legs and this could partially explain the higher percentage of scaly surface and collarette sign among KS nodules. Conversely, 4/9 plaques and 1/2 patches were observed on the legs.

**Table 3:** Frequency of ultrasonographic and Doppler-mode features in KS lesions and in non-KS lesions.

Type of KS Lesion (n)	Hypoechoic Band/Nodule, n (%)	Doppler-Mode Signal, n (%)	Vascularization Type I–IV, (n, %)
KS lesions (total = 32)	30 (93.7)	21 (65.6)	-
Patch (2)	-	-	-
Plaque (9)	9 (28.1)	1 (3.1)	-
Nodule (21) Single (8) Multiple (5) Hyperkeratotic (3) On plaque (3) Deep (2)	21 (65.6) 8 (25) 5 (15.5) 3 (9.4) 3 (9.4) 2 (6.2)	20 (62.5) 8 (25) 5 (15.5) 2 (6.2) 3 (9.4) 2 (6.2)	Type I (1, 4.8), Type II (16, 76.2), Type III (0, 0), Type IV (4, 19) Type II (6, 75), Type IV (2, 25) Type II (3, 60), Type IV (2, 40) Type I (1, 33.3), Type II (2, 66.7) Type II (3, 100) Type II (2, 100)
Non-KS lesions (total = 9) Cherry angioma	9 (100) Yes	5 (55.56) No	Type I (4, 44.5), Type II (2, 22.2), Type III (0, 0), Type IV (3, 33.3) 1
Venous Lake	Yes	No	1
Superior Lip Venous Lake	Yes	No	1
Subcutaneous Venous Lake	Yes	No	1
Glomus Tumour	Yes	Yes	2
Pyogenic Granuloma	Yes	Yes	2
Angiosarcoma	Yes	Yes	4
Nodular Melanoma Merkel Cell Carcinoma	Yes Yes	Yes Yes	4 4

## Data Availability

The data presented in this study are available on request from the corresponding author. The data are not publicly available due to restrictions for privacy.
